# Health care provider payment reforms in African states of the Commonwealth—a scoping review

**DOI:** 10.3389/fpubh.2025.1446497

**Published:** 2025-06-18

**Authors:** Costase Ndayishimiye, Christoph Sowada, Katarzyna Dubas-Jakóbczyk

**Affiliations:** ^1^Doctoral School of Medical and Health Sciences, Jagiellonian University Medical College, Krakow, Poland; ^2^Department of Health Economics and Social Security, Institute of Public Health, Jagiellonian University Medical College, Krakow, Poland

**Keywords:** healthcare provider, strategic purchasing, payment reform, commonwealth, Africa

## Abstract

**Introduction:**

Healthcare provider payment reform is a key element of strategic purchasing to improve health system efficiency, equity, and quality. Although such reforms are well documented in high-income countries, evidence in low- and middle-income countries—particularly in sub-Saharan Africa—remains limited and fragmented. This scoping review aimed to identify, map, and systematize recent literature on provider payment reform for strategic purchasing and the factors influencing these reforms in 21 African Commonwealth countries.

**Methods:**

The review followed the scoping review methodological guidelines of Peters et al. and was reported using the PRISMA-ScR checklist. Studies were retrieved from scientific databases and supplemented with gray literature. Factors influencing the reforms were analysed using a health policy framework covering context, content, process, and actors.

**Results:**

Thirty-five full-text publications were included (29 empirical studies, four technical reports/policy briefs, and two reviews). The evidence spans eight countries, with six focusing on performance-based financing (PBF). Reforms often added new payment methods to existing ones (62.85%, *n* = 22/35), replaced existing methods (typically fee-for-service (FFS) with capitation in primary care (28.57%, *n* = 10/35)), or adopted mixed methods (37.14%, *n* = 13/35), with blending FFS and capitation being the most common. Multiple factors influenced different reform dimensions. Political inattention and inadequate policy, legal, and regulatory frameworks hindered the reform context. Reform content depended on clear core elements such as performance indicators, guidelines, tariffs, financial rewards, and provider autonomy. Factors such as a lack of reform piloting, chronic underfunding, fragmented funding flows, and inadequate monitoring and evaluation mechanisms hindered the reform process. The actor dimension was impacted by a lack of a holistic approach to stakeholders and limited stakeholder capacity to implement reforms.

**Discussion:**

Current evidence for implementing provider payment reforms remains limited—concentrated in a few countries and often focused on specific reform types or evaluations from a single perspective. Future studies could focus on more comprehensive reform evaluations, incorporating multistakeholder perspectives and links with other elements of strategic purchasing.

**Systematic review registration:**

https://archive.org/details/osf-registrations-vs4fd-v1.

## 1 Introduction

African leaders have demonstrated a strong commitment to advancing Universal Health Coverage (UHC), as reflected in key policy documents such as the Africa Health Strategy (2007–2015, extended to 2016–2030) and the Addis Ababa Call to Action on UHC in 2019 (1). These documents highlight the continent’s collective efforts to ensure equitable access to quality healthcare services for all citizens. Despite this widespread support, numerous challenges persist in pursuing UHC within resource-limited settings (2).

Strategic purchasing is recognized as a crucial health financing policy approach aimed at optimizing the use of limited resources to progress toward UHC (2, 3). This approach directs funds to priority populations, interventions, and services on the basis of evidence and health needs. Strategic purchasers in healthcare, such as the Ministry of Health, Social Health Insurance Fund, or local authorities, make deliberate decisions on the basis of five key areas: (1) coverage—determining for whom healthcare services should be purchased; (2) benefit package—deciding which services to purchase; (3) contracting—selecting providers; (4) quality—ensuring the quality of services; and (5) provider payment—determining the payment methods and prices for providers (4).

Previous studies have assessed the progress of various aspects of strategic purchasing in some African countries purchasing, including benefit design for improving access to priority services (e.g., high-value services such as reproductive and family planning; maternal, neonatal, and child health services) and stakeholder contracting arrangements (5–8). However, evidence on provider payment reforms in low- and middle-income countries (LMICs)—particularly in sub-Saharan Africa—remains limited, fragmented, and largely descriptive (9). Many existing studies focus on specific schemes such as performance-based financing (PBF), often within individual country contexts (10–13). Systematic analyses that explore broader patterns and influencing factors across LMICs are rare (9, 14).

In contrast, evidence from high-income countries (HICs) shows that reforming healthcare provider payment schemes is a popular policy tool used to improve efficiency, quality, accountability, and overall health system performance (15–19). Payment schemes are designed to influence healthcare providers’ behaviors, thereby playing a crucial role in strategic health purchasing. Common provider payment methods include: fee-for-service (FFS), where providers are paid per individual service delivered; capitation, which pays providers a fixed amount per patient over a set period regardless of service use; and performance-based financing (PBF), which links payments to the achievement of specific quality or service indicators. Each method offers distinct incentives and trade-offs—FFS can encourage an oversupply of care, capitation incentivizes efficiency but risks underprovision, while PBF emphasizes results but may increase administrative complexity (15, 20). Nevertheless, implementing such reforms successfully is challenging and often influenced by a mix of diverse barriers and facilitators (17–19).

This review aimed to identify, map, and systematize recent literature (published within the last decade) on provider payment reform for strategic purchasing and the factors influencing these reforms in 21 African Commonwealth countries (Botswana, Cameroon, Gabon, Gambia, Ghana, Kenya, Eswatini, Lesotho, Malawi, Mauritius, Mozambique, Namibia, Nigeria, Rwanda, Seychelles, Sierra Leone, South Africa, Togo, Uganda, Tanzania, and Zambia). On the basis of general objectives of the scoping method (21), we examined the breadth of existing evidence, identified potential research gaps, and formulated implications for future studies.

## 2 Methods

The study followed the scoping review methodological guidelines of Peters and colleagues (21, 22), which included five steps: defining review questions, identifying relevant literature, selecting evidence, extracting evidence, and analyzing data. The results were reported via the PRISMA-ScR checklist (23), and the study protocol was registered with the Open Science Framework (24).

### 2.1 Defining research questions

The specific questions guiding the review were as follows:

1. What type of evidence is available? (study country, publication year, study type, study objective).
2. What type of payment method was analyzed (type of method, type of change)
3. What type of healthcare providers were involved (e.g., primary care vs. hospital)?
4. What factors (obstacles and facilitators) influenced the reform?

### 2.2 Identifying relevant literature

Three scientific databases—PubMed, Scopus, and Web of Science—were searched for empirical studies. The search strategy was iteratively developed and conducted using multiple synonyms of “healthcare provider” AND “payment” AND “country” in titles and abstracts. Complementary searches included Google and gray literature on relevant organization websites, such as the Strategic Purchasing Africa Resource Centre (SPARC), the WHO via WHO African Region, the World Bank, Responsive and Resilient Health Systems (RESYST), and Health Finance and Governance (HFG) country publications. The reference lists of the included publications were manually searched for additional studies. Details of the search strategy and records for each data source are provided in Supplementary Tables S1–S6. The searches were conducted between June and July 2023.

### 2.3 Selecting evidence

The publications were selected in two stages: screening abstracts and evaluating full texts on the basis of predefined inclusion and exclusion criteria (Supplementary Table S7). Studies were included if they: (1) focused on provider payments within strategic purchasing; (2) were peer-reviewed empirical studies, policy briefs, theoretical papers, technical reports, books/chapters, or theses; (3) focused on an African Commonwealth country; (4) were published between 2013 and 2023; and (5) were available in English. Studies were excluded if they: (1) did not focus on healthcare provider payment within strategic purchasing (e.g., focused on social insurance schemes, community financing, cost recovery, medication payments, or informal caregiving); (2) were not full-text publications (e.g., conference abstracts); (3) focused on non- African Commonwealth countries; (4) were published before 2013; or (5) were in other languages.

Two independent researchers (CN and KDJ) conducted the title and abstract screening phase, achieving an agreement level above 80%. The full-text evaluation was performed by one researcher (CN) and reviewed by KDJ. Mendeley and Rayyan software were used for data management.

### 2.4 Data extraction

Data extraction tables were created using MS Excel and were tailored to specific research questions. A single researcher (CN) conducted the extraction, which was reviewed by another researcher (KDJ) and further by all coauthors during the draft and final manuscript review stages.

### 2.5 Data analysis and reporting

The study employed inductive thematic analysis to analyze qualitative data, which was then coded for quantitative summaries and tabulated. The paper types were classified into four categories: empirical studies (original, based on primary data, published in peer-reviewed journals), discussion/policy papers (published in peer-reviewed journals), literature reviews (published in peer-reviewed journals), and technical reports/policy briefs (e.g., policy briefs published by advocacy organizations). For payment methods reforms, the OECD classification (15) was used to code whether the reform modified an existing payment method, introduced an additional method, or replaced it with a new method.

Factors influencing reforms were deductively classified using the health policy framework (25–27), which consists of CONTEXT (systemic factors, e.g., political, economic, and cultural influences), CONTENT (detailed elements of a reform), PROCESS (creation, communication, implementation, and evaluation of the reform), and ACTORS (participants in policymaking: individuals, organizations, groups, and the government).

## 3 Results

### 3.1 Search results

**Figure 1:**
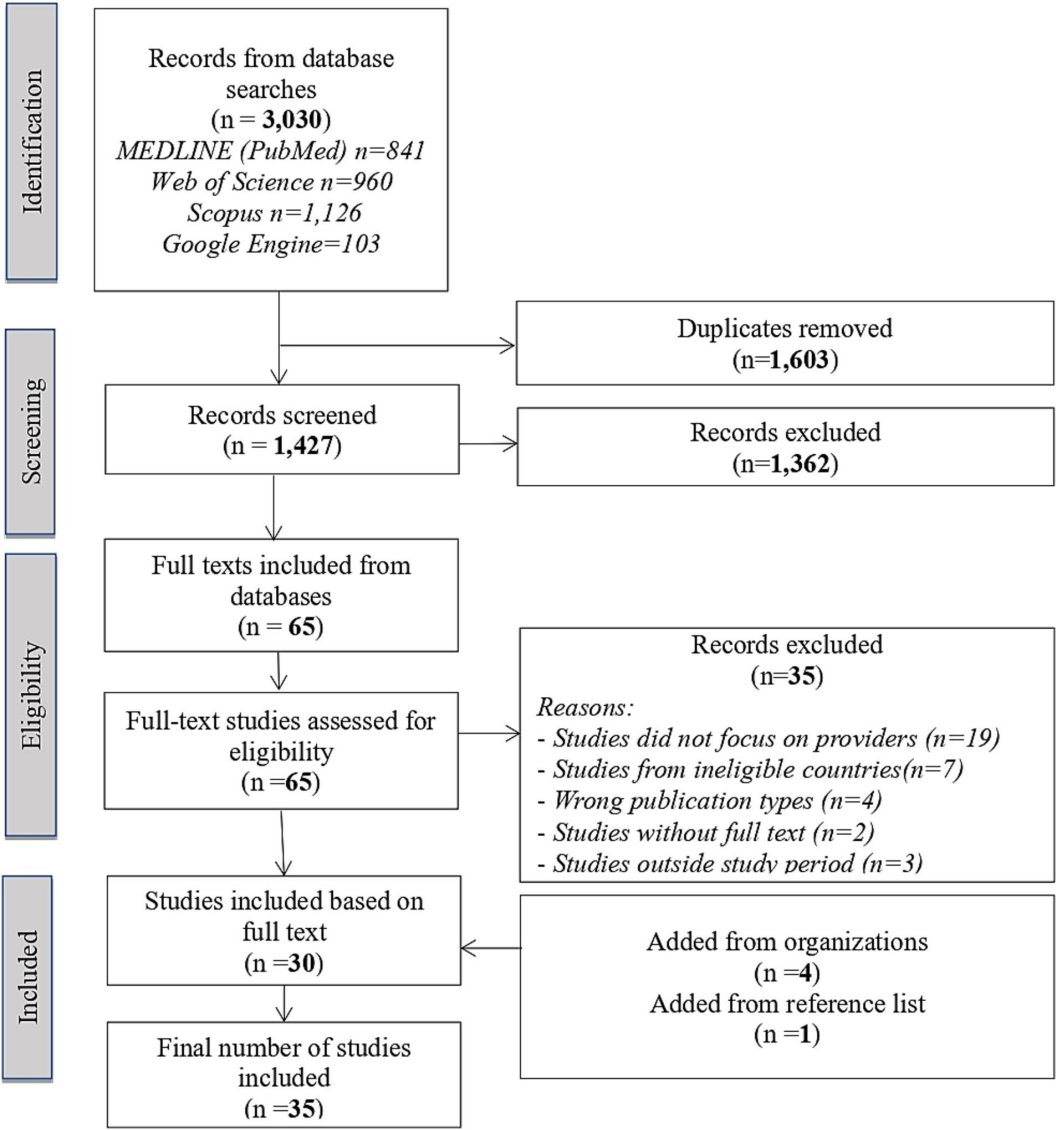
PRISMA flowchart of the results.

The database searches yielded 3,030 records, with 1,603 duplicates. After screening 1,427 titles and abstracts, 65 full texts were reviewed, and 30 met the inclusion criteria. Most studies (*n* = 19) were excluded because they did not focus on healthcare providers. Four articles from organizations and one from reference lists were included, totaling 35 publications for the final analysis. Figure 1 shows the PRISMA flowchart, and Table 1 lists all included studies by country and relevant details aligned with the study questions.

**Table 1:** Overview of the characteristics of the included studies by country.

Country	References	Year	Study type a*	Study aims b*	Reform objectives	Reform content			Factors influencing reforms (blue = facilitator, red = barrier)			
						Pay method	Change c*	Provider	Context	Content	Process	Actors
Cameroon	SPARC (48)	2021	tec/pb	3	Reduce maternal and child mortality through targeted healthcare services.	FFS (with additional incentives via P4P)	add	Multiple	- Presence of legal mandate for strategic purchasing (54). - Lack of relevant policy frameworks (54). - Highly centralized administration reduced purchasers’ autonomy to influence reform objectives (54).	- Well-defined service delivery standards were integral in provider contracting and renewals (11, 48, 54). - Regular revision of indicators (54). - Providers given financial and managerial autonomy (48, 54). - Presence of clear methods for setting purchaser’s budget and tracking expenses (48). - Multiple purchasers with varied in institutional arrangements (48, 54).	- Regularly monitored providers for production quantity and quality (54). - Poor monitoring and information systems hindered proactive detection of provider misconduct (11, 48, 54). - Lack of harmonized funding flows and payment systems across different schemes (11, 48, 54). - Underfunding, reliance on donor funding (e.g., World Bank support for PBF) (48, 54). - Delays/irregular payments to providers (11). - Publicity, awareness, and education gaps on payment reform (e.g., hindered PBF reform efforts) (11).	- Multiple stakeholders— Government, public, private, community-based organizations, and external agencies (donors) —were engaged (11, 48, 54). - Lack of measures to balance stakeholder powers (54).
	Sieleunou et al. (54)	2021	lit rev	3	Link provider payments and funding to service quantity and quality, focusing on maternal and child health.	P4P	add	Multiple				
	Nkangu et al. (11)	2022	emp-QL	1	Prioritize maternal and child health services delivery.	P4P	add	Multiple				
Ghana	Aboagye (55)	2013	emp-mix	1	Control costs, simplify claims processing, enhance efficiency, improved forecasting and budgeting.	Capitation (replacing FFS)	rep	PHC	- Capitation policy sparked political debates, heavily influenced by MPs, thereby garnering attention (32, 58). - The chosen Ashanti region for piloting capitation was home to major opposing political party, raising political suspicion (32). - Politicians, disguised as pressure (anti-capitation) groups, allegedly exploited their hidden interests to gain political points and discredit the government (58).	- Clear indicators (e.g., quality), coherent guidelines and management arrangements ensured effective financial reporting and accountability during reform implementation (e.g., capitation) (32). - Ensuring clear tariffication (e.g., G-DRG system included 600 tariff criteria for outpatient and inpatient services, determined by costing and provider negotiations) (33). - Insufficient data/evidence (e.g., base per capita rate applied to pay providers lacked data for adjusting coefficients) (32). - Inadequate reimbursement rates (e.g., low rates for capitation) hindered reform by causing provider dissatisfaction and unrest (28, 32, 57, 59). - Lack of trust in NHIA’s timely payments weakened capitation cost containment efforts (32).	- Piloting reforms (e.g., capitation in Ashanti Region) (28–32, 55–58). - Training modules for providers were developed on financial and other management changes under the capitation payment system (32). - A monitoring and evaluation system was designed to measure the impact of capitation on trends in quality of care, utilization, access to healthcare, cost containment, and provider experience (32). - Failure to pilot and prospectively evaluate G-DRG exacerbated cost escalation, ultimately causing reform failure (28, 57). - Misleading advertising/ negative publicity/lack of effective public education (e.g., the media spread misinformation about capitation payments, misleading providers and causing opposition) (28–30, 32, 55, 56). - Payment delays/irregular rates to providers (31–33, 55, 57, 59). - Chronic underfunding, reliant on donor funds (e.g., capitation tied to World Bank funds) (31, 32, 56, 57, 59). - Fragmented health service delivery systems (33, 57). - Insufficient information/IT tools (e.g., limited E-claim systems hindered claims processing; most work still relied on manual processes) (31, 33, 57). - Irregular fund flows with unpredictable amount (57). - Providers opposed capitation payment due to their prior favoritism toward G-DRG and FFS (they wanted to evade cost-sharing roles included in capitation) (30, 32).	- A multi-stakeholder provider payment mechanism technical steering committee (PPM TSC), comprising experts in health financing, implementation and authorities, was formed to design the capitation policy and plan its implementation (32, 58). - The capitation payment reform involved mapping capacities of all facilities, exposing significant capacity differences. Facilities meeting standards operated independently, while those lacking capacity formed groups in order to operate (32). - Poor public participation (lack of involvement of interest groups from the general public, e.g., care seekers/patients’/community organizations) (29, 55).
	Agyei-Baffour et al. (56)	2013	emp-QN	2	Contain costs, share financial risks, implement managed competition and enhance patient choice.	Capitation (replacing FFS)	rep	PHC				
	Atuoye et al. (58)	2016	emp-QL	1	Contain costs: strengthen claims processing to curb fraud.	Capitation (replacing FFS)	rep	PHC				
	Anyona (59)	2018	tec/pb	3	Contain costs.	Capitation (replacing FFS)	rep	PHC				
	Andoh-Adjei et al. (28)	2018	emp-QN	1	Control utilization and contain costs of claims.	Capitation (replacing FFS)	rep	PHC				
	Abduali et al. (29)	2019	emp-QN	2	Contain costs and share risks.	Capitation (replacing FFS)	rep	PHC				
	Andoh-Adjei et al. (30)	2019	emp-QN	2	Control cost escalation.	Capitation (replacing FFS)	rep	PHC				
	Aikins et al. (31)	2021	emp-QL	2	Contain costs and reduce fraud in claims submission.	Capitation (replacing FFS)	rep	PHC				
	Amporfu & Arthur (32)	2022	tec/pb	2	Control cost escalation.	Capitation (replacing FFS)	rep	PHC				
	Agyepong et al. (57)	2014	emp-mix	2	Reduce cost escalation and solve claims processing inefficiencies.	Mixing (G-DRG and FFS).	add	Hospital				
	Amporfu et al. (33)	2022	emp-QL	3	Control cost escalation.	Mixing (DRG and FFS).	add	Hospital				
Kenya	Munge et al. (34)	2018	emp-QL	1	Incentivize efficiency, service quality, and promote equitable access.	Mixing (capitation, case-based payments, and FFS)	add	Multiple	- Weak regulatory and policy framework (e.g., The NHIF Act of 1998 provides guidelines for mandates and functions but does not address strategic purchasing issues like provider payment methods) (34–36, 39).	- Unclear rationale for designing payment systems (e.g., capitation was theoretically chosen to mitigate overservicing risks associated with FFS and per diem payments) (34). - Providers resisted new payment forms due to concerns over payment rates estimation (they perceived capitation rates as insufficient for covering actual care costs) (34, 36–40). - Weak provider accountability mechanisms (34, 35). - Inadequate quality assurance mechanisms (e.g., reliance on facility utilization of MoH standards and treatment guidelines despite hospitals’ evidence indicating poor adherence to these guidelines.) (34–37, 39). - Reduced provider financial autonomy limited their decisions, power, and demotivated them (35, 37, 40).	- Implementing measures to mitigate payment incentives’ unintended effects, such as regular facility visits, capped claims, staff fraud training, and establishing risk investigation units (34). - Lack of required resources (insufficient resources allocated for meeting service delivery demands) (34, 35). - Inadequate monitoring (lack of framework and reporting structures to monitor provider performance and adherence to standards) (34, 35, 39, 40). - Inadequate complaints and feedback mechanisms (34–36, 40). - Provider payment delays and unpredictability (35, 37, 38, 40). - Insufficient health information systems (reliance on paper-based records due to limited electronic systems, computer shortages, and frequent network failures) (35, 37, 39). - Fragmented/poor coordination between health and financing structures (multiple payment mechanisms lacking coherence across different schemes) (36, 39, 40).	- Poor public participation (lack of involvement of interest groups from the general public, e.g., care seekers/patients’/community organizations) (34–36).
	Mbau et al. (35)	2018	emp-QL	3	Encourage efficiency and service quality.	Mixing (line-item budgets and salaries)	noch	Multiple				
	Munge et al. (36)	2019	emp-QL	1	Improve efficiency, control cost, enhance service quality and access.	Mixing (FFS and capitation)	add	Multiple				
	Obadha et al. (37)	2019	emp-QL	2	Improve efficiency, quality, and utilization of needed services.	Mixing (FFS and capitation)	add	Multiple				
	Obadha et al. (38)	2020	emp-QN	2	Enhance service quality and efficiency.	Mixing (capitation and FFS)	add	Hospital				
	Kazungu et al. (39)	2021	emp-QL	3	Incentivize providers to deliver quality services, efficiently, and equitably.	Mixing (capitation, case-based payments, FFS)	mod, add	Multiple				
	Kabia et al. (40)	2022	lit rev	3	Improve efficiency, equity, access, and quality of care.	Mixing (capitation, case-based payments, FFS)	mod, add	Multiple				
Mozambique	Schuster et al. (12)	2018	emp-mix	2	Improve HIV services, reduce mother-to-child HIV transmission (PMTCT), and enhance maternal/child health (MCH) services.	P4P	add	Multiple	- PBF scheme gained significant political support, especially at the district level (12).	- Health facilities were given autonomy to manage funds, prioritize their specific issues, and address implementation barriers independently (12).	- Although the supervisions were well-organized and inspiring, they led to excessive leadership duties (managers in the fields of mother and child health complained that PBF made them more invested in roles as supervisors) (12). - Delays in PBF disbursements, due to internal processing and facility management issues, including leadership transitions, caused frustration among providers and administrators (12). - Insufficient funds (e.g., stock-outs of essential equipment like HIV tests and drugs) (12).	- Engaged key stakeholders (e.g., providers, government) (12). - Providers’ involvement in PBF design and implementation fostered feelings of ownership and fulfillment of motives like autonomy, feeling valued, and competence demonstration (12). - Poor public participation (lack of involvement of interest groups from the general public, e.g., care seekers/ patients’/ community organizations) (12).
Nigeria	Ezenduka et al. (41)	2022	emp-QL	3	Control costs, improve efficiency, and quality of services.	Mixing (capitation and FFS)	add	Multiple	- Presence of policy, legal, and governance structures and frameworks for strategic purchasing (41–43). - NHIS encountered governance obstacles, including political interference compromising financial autonomy and decision-making power for effective purchasing (42).	- Use of well-defined benefit package, metrics, and guidelines (e.g., NHIS standardized treatment and quality protocols) (41, 43). - Provider contracting involved meeting service and target criteria; noncompliance with personnel and facility standards led to nonrenewal (42, 43). - Payment rates were established via actuarial studies (43). - Weak accountability mechanisms (lack of structures to monitor and evaluate provider performance) (41, 43). - Restricted financial autonomy hindered provider service prioritization and access to financial resources (41).	- Providers received training on reform activities (e.g., using implementation guidelines and reporting quantitative data) (41). - Inadequate monitoring of providers and purchasers (41, 42). - Insufficient health information systems (inadequate technology to collect relevant information on provider activities for evidence-based planning and decision-making; health-related information remained predominantly paper-based; and providers lacked adequate electronic systems due to a lack of computers) (41, 42). - Lack of feedback and complaints mechanisms (41, 42). - Inadequate budget allocation/chronic underfunding, reliance on donors (41, 43). - Providers faced frequent delays and inadequate payments, resulting in service rationing and charging user fees for supposedly free services (41–43). - Fragmented funding flows through different schemes (42, 43).	- Involvement of multiple stakeholders: providers, government authorities, and donors (41). - Poor public participation (lack of involvement of citizens or their associations) (41).
	Ezenwaka et al. (42)	2022	emp-QL	3	Contain cost, enhance efficiency and quality care.	Mixing (capitation and FFS)	add	Multiple				
	Onwujekwe et al. (43)	2022	emp-QL	3	Contain costs, improve efficiency and service quality.	Mixing (capitation and FFS)	add	Multiple				
Rwanda	Binagwaho et al. (44)	2014	emp-QN	1	Enhance maternal and child health care service quantity and quality services.	P4P	add	Multiple	- Rwanda integrated PBF policy into its nationwide development policies and plans (e.g., millennium development goals, etc.) (44–46). - Established regulatory framework that supports strategic purchasing (46). - In 2015, Rwanda’s government restructured major schemes to consolidate management and create an efficient, sustainable purchaser-provider split (46). - In 2006, decentralization health reforms granted autonomy to public health facilities, facilitating reforms like PBF (46).	- Clearly defined indicators determined based on national priorities and service delivery protocols of the MOH (46). - In 2014, the MOH tied PBF incentives to hospital accreditation, motivating managers to pursue it and improve service quality (46). - Overlapping mandates and functions between key institutions and actors (similar purchasing functions performed by multiple institutions) (46).	- PBF piloting before nationwide expansion (44–46). - PBF indicators’ weight and costs regularly reviewed transparently using evidence-based processes (46). - PBF contracting was tied to the country’s Imihigo performance contracting process (46). - Regular internal and external PBF audits (46). - Providers given autonomy to manage revenue generated (46). - Availability of various performance monitoring mechanisms and tools (46). - Adequate deployment of information systems (e.g., DHIS2 software, Mutuelle Membership Management System (3MS), e-payment technologies, EMRs) (46). - Limited interoperability among deployed health information systems hindered timely decisions (46). - Lack of a biometric fingerprint system and accurate, real-time data hindered efforts to detect fraud and abuse (46). - Limited funding (despite the government budget being the main source of PBF funds, its sustainability remained a major challenge) (46).	- Involvement of multiple stakeholders: government, private/public providers, insurers, and citizen representatives (44–46). - Established community health committees with community representatives at public health facilities and district health units –(46).
	Ngo et al. (45)	2017	emp-QN	1, 2	Incentivize health facilities based on performance in maternal health, child health, family planning, HIV/AIDS, and overall facility quality.	P4P	add	Multiple				
	Umuhoza et al. (46)	2022	emp-mix	3	Incentive providers to focus on maternal and child health, HIV, tuberculosis, and child stunting.	P4P	add	Multiple				
Tanzania	Manongi et al. (13)	2014	emp-QL	2	Improve service quality.	P4P	add	PHC	- PBF garnered broad political support, notably from the Ministry of Health and Social Welfare (MoHSW) (13). - Absence of an officially established national policy and guidelines for PBF in healthcare (13).	- Granting provider autonomy enabled them to be creative and enhance care quality (13). - Use of standard treatment guidelines provided by the MoH (49). - Transparent provider contracting and accountability mechanisms (49). - Price/fee rates were determined based on a comprehensive review of policy documents, actuarial valuation, costing studies, and expert advice (49). - The price list was periodically reviewed and adjusted to meet up-to-date requirements (49).	- Piloting reform (e.g., PBF in Pwani region) (13, 47). - Health personnel were trained on PBF principles to enhance their general knowledge and skills in PBF programs (13). - Periodic evaluation of provider performance data (30). - Implementing a routine monitoring through a health information system facilitated effective oversight of healthcare service delivery, including registration, claims processing, referrals, and broader population healthcare (49). - Insufficient funding, reliance on development partners, loans, and donors (13, 47, 49). - Payment delays to service providers (13). - Fragmented financing systems (disjointed payment mechanisms across diverse schemes) (47, 49).	- Involvement of multiple stakeholders: public, private, faith-based, and donors (13, 49). - Community involvement (community participation on the PBF governing board at facility level enhanced communication between the community and health facilities) (13). - Most health facilities experienced deficiencies in both medical and nonmedical human resources (13).
	Binyaruka and Anselmi (47)	2020	emp-QN	1	Enhance maternal and child health services.	P4P	add	Multiple				
	Kuwawenaruwa et al. (49)	2022	emp-QL	3	Enhance service access, efficiency, and care quality.	Mixing (capitation and FFS)	mod, add	Multiple				
Uganda	Ekirapa-Kiracho et al. (50)	2017	emp-QN	3	Enhance care quality, efficiency, and quantity, prioritizing maternal and child mortality prevention.	Mixing (FFS, capitation, and line-item budgets).	add	Multiple	- In 2017, a national PBF framework was launched (51). - To enhance the complementarity of roles, the government introduced the National Policy on Public Private Partnership in Health (PPPH), subsidizing accredited private health providers, primarily religiously affiliated (51). - Inadequate legislation to support strategic purchasing (50, 51). - Centralized health system bureaucratic procedures rendered lower-tier facilities nonautonomous (50, 51).	- Clearly defined priority interventions, package, and performance indicators (linking bonuses to results) (50, 51). - Reward and sanction systems intended to enhance appropriate provider behaviors lacked clarity and remained inactive in many facilities (50). - Informal pricing undermined transparency and accurate cost estimation (50). - PBF was linked with quality indicators, yet target specification was still developing in Uganda (51).	- Insufficient funding stemming from inadequate domestic financing and dependence on development partners via on- and off-budget support mechanisms (50, 51). - Challenges arising from existing health system inadequacies, including insufficient healthcare personnel, low morale absenteeism, and inadequate infrastructure (50). - Lack of provider training and supervision (50). - Fragmented purchasing systems, with multiple concurrent financing systems (51). - Failure to coordinate actions, especially between purchasers and patients (e.g., lack of feedback and social accountability mechanisms) (51).	- Engaging diverse stakeholders: government, donors, and insurance schemes (predominantly private commercial and community-based) (50, 51). - Poor public participation (lack of involvement of citizens or their associations) (51).
	Ekirapa-Kiracho et al. (51)	2022	emp-QL	3	Provide key services for maternal and child health, and communicable diseases like malaria.	P4P	add	Multiple				
Multicountry (Ghana, Tanzania)	Yé et al. (52)	2014	emp-QL	2	Enhance quality of maternal and neonatal health care provision	P4P	add	Multiple	No findings.	- Lack of transparency in the selection of P4P indicators (e.g., ambiguity in provider performance measurement criteria) (52).	- Training providers for P4P reform enhanced their skills and task performance capabilities (52). - Mobilizing local resources to enhance sustainability of the scheme (52). - Regular supervision alerted providers to errors and ways to enhance their performance (52). - Concerns regarding the workload necessary for managing P4P schemes (52). - Insufficient funds, heavy dependence on donors (52). - Providers hesitated to engage due to incentive misalignment and doubted managers’ capability to deliver P4P schemes (52).	- Involving healthcare providers in P4P scheme design enhanced their buy-in and reform endorsement (52).
Multicountry (Ghana, Mozambique)	Cashin et al. (53)	2018	tec/pb	3	Contain costs and expand access to priority services – maternal and child health care.	Capitation, P4P	add	Multiple	- Establishing clear institutional roles and relationships, both desired and actual, to make it possible to identify who has the authority for which strategic purchasing policies and is accountable for implementing them (53).	- Mapping existing roles and relationships for strategic purchasing and identifying gaps or conflicts provided a solid foundation for effective planning (53). - A well-structured activity plan enabled informed decision-making and created an environment that supported reaching reform objectives (53). - Granting autonomy for health facilities to hire, fire, and assign staff improved reform management and enhanced cost efficiency (53). - Using disparate payment methods increased financial strain and hampered efforts to achieve efficiency (53).	- Presence of systems facilitating strategic purchasing process, including provider accreditation, empanelment contracting, and performance monitoring, proved instrumental in achieving reform objectives (53). - Targeted training to equip key stakeholders with the necessary knowledge and skills to carry out their roles (e.g., a trainer training program was developed for capitation reform in Ghana involving over 600 district NHIS staff) (53). - Lack of sufficient information systems/IT tools (e.g., Ghana’s capitation lacked sufficient e-claims systems to automate claims data; providers continued to submit claims using Excel) (53).	- Involvement of multiple key actors (purchasers, providers, regulators, donors) (53). - Ensuring sufficient technical capacity for stakeholders to carry out their roles and responsibilities (53). - Poor public participation (lack of involvement of citizens or their associations) (53).

a*(emp-QL: empirical study-qualitative; emp-QN: quantitative; or emp-mix: mixed; tec/pb: technical report/policy brief; lit rev: literature review). b*(1 – payment reform evaluation - from the system perspective; 2 – payment reform evaluation from the provider perspective; 3 – general overview of the strategic purchasing progress including payment method analysis/description). c*(mod: Modifying the existing payment method; add: Implementing an additional payment method; rep: Replacing the existing method with the new one; noch: no change).

### 3.2 Overview of publications

The 35 studies included (11–13, 28–59) were from eight countries. Of these, 29 were empirical studies, four were technical reports/policy briefs (32, 48, 53, 59), and two were reviews (40, 54). One review was a scoping review mapping progress in strategic health purchasing in Cameroon (54), and the other was a narrative review assessing health purchasing reforms’ effects on equity, access, quality of care, and financial protection in Kenya (40). Empirical studies primarily evaluated specific experiences with payment reforms, notably pay-for-performance [P4P, *n* = 12/35, also known as results-based financing (RBF) or performance-based financing (PBF)] and capitation (*n* = 10/35). Studies utilized qualitative (*n* = 16/35), quantitative (*n* = 9/35), or mixed methods (*n* = 4/35) to assess these reforms. Reform assessment often emphasized specific stakeholders’ perspectives, including providers’ experiences, opinions, and preferences (31.42%, *n* = 11/35). Examples include Ghana’s capitation (30, 31, 56, 57), Kenya’s capitation and fee-for-service (FFS) (37), and PBF in Mozambique (12) and Rwanda (45). Some evaluations took a system perspective (22.85%, *n* = 8/35), assessing payment methods’ effectiveness in achieving broader healthcare system objectives [for example, the evaluation of PBF strategies to improve maternal health service access and utilization in Cameroon (11)]. In some cases, evaluations aimed to draw lessons from healthcare provider payment reforms for the entire health system. An example is the evaluation of capitation in primary healthcare (PHC) in Ghana, which aimed to inform a nationwide rollout (55). Evaluations could also compare achievements before and after payment reform, as observed in Tanzania’s study on technical efficiency before and after the P4P scheme (47).

### 3.3 Payment methods reforms

Reforms in many countries frequently centered on adding new payment methods to existing ones (62.85%, *n* = 22/35), with P4P being the most commonly adopted method to bolster the strategic purchasing of specific curative, preventive, and promotional services (Table 1). Many reforms concentrated particularly on maternal and child health. Primary prevention efforts prioritized vaccinations such as childhood immunizations (e.g., measles) and maternal tetanus vaccinations during prenatal care, as seen in Rwanda (46), Tanzania (13), and Cameroon (11). Prevention measures aimed at controlling infectious diseases such as HIV and tuberculosis were also noted in Mozambique (12), Cameroon (54), and Rwanda (44, 45). In 37.14% (13/35) of the studies, countries added methods with the intention of using mixed methods to pay providers, predominantly combining FFS and capitation. This approach was evident in countries such as Kenya (34, 36–38), Uganda (50), Tanzania (49), and Nigeria (41–43). Other reforms were implemented to completely replace existing payment methods, notably replacing FFS with capitation in PHC, prominently in Ghana (28–32, 55, 56, 58, 59). Ghana introduced capitation in 2012 to contain costs, share financial risk, enhance competition, and improve efficiency and claims processing after previous methods such as FFS and diagnosis-related grouping (G-DRG) were ineffective in addressing these challenges (32, 55, 56).

### 3.4 Types of providers involved

Certain payment reforms targeted specific providers, such as replacing FFS with capitation in PHC. However, most reforms, such as PBF, were broadly applied across various provider categories, including PHCs, hospitals, and/or specialty care (11, 46, 48, 51, 54). Both public and nonpublic sectors were sometimes included, as seen in Kenya (37) and Tanzania (49), where capitation and FFS were applied to public, private, and charity providers, and in Cameroon (11, 48), for PBF.

### 3.5 Factors influencing payment reforms

In the surveyed countries, various interconnected factors impacted different reform dimensions (Table 1). The reform context was frequently shaped by political will, policies, legal frameworks, and governance structures for strategic purchasing practices. Political neglect often led to superficial endorsement of reforms without sustained commitment, resulting in inconsistent implementation (32, 42, 58). Inadequacies in legal and regulatory frameworks hindered the effective operationalization of reforms, resulting in implementation inefficiencies and gaps (13, 34–36, 39, 50, 51, 54).

Reform content factors stemmed from essential elements such as guidelines, performance indicators, tariffs, financial incentives, and providers’ autonomy over finances. These were important to ensure clarity, consistency, and alignment with reform objectives. Guidelines guided reform implementors (32, 41, 43, 46, 49–51). Unclear indicators hindered many reforms, but good examples were observed in countries like Rwanda (46), Cameroon (54), Ghana (32), Nigeria (41, 43), and Uganda (50, 51). Transparent tariffs provided fair incentives (33, 43, 48, 49), whereas financial autonomy allowed providers to use resources flexibly, responsively, and responsibly [e.g., PBF programs in Camerron (48, 54), Mozambique (12), and Tanzania (13)].

Several factors impacted the reform process dimension, with top barriers stemming from the absence of reform piloting, chronic underfunding, fragmented funding flows, and inadequate monitoring and evaluation mechanisms. Piloting reform helped identify implementation challenges and informed its redesign before a nationwide rollout [e.g., capitation in Ghana (55) and PBF programs in Rwanda (44–46) and Tanzania (13, 47)]. Piloting proved essential for detecting and addressing potential issues early. Chronic underfunding crippled the ability to sustain long-term reform initiatives. Payment reform in various countries suffered heavy dependency on donor funds (13, 31, 32, 41, 43, 47–52, 54, 56, 57, 59). Fragmented funding flows with often multiple payment systems further exacerbated these issues by creating inefficiencies and misallocations of resources (11, 36, 39, 40, 42, 43, 47–49, 51, 54, 57). Inadequate monitoring and evaluation mechanisms led to a lack of accountability and transparency, impeding the ability to measure progress and make necessary adjustments (11, 34, 35, 39–42, 48, 51, 54).

Finally, the reform actor dimension was frequently impacted by barriers associated with a lack of a holistic approach to stakeholders and inadequate stakeholder capacity to perform reform tasks. Reforms and involved stakeholders varied within and between countries. Frequent actors can generally be grouped into government, purchasers, healthcare providers (including provider groups), and the general public. Notably, in most reforms, the general public, such as citizens or patients and their associations, was commonly overlooked (12, 29, 34–36, 41, 51, 53, 55).

## 4 Discussion

Evidence suggests that since 2013, only eight of the 21 African Commonwealth countries have implemented healthcare provider payment reforms. This underscores a scarcity consistent with previous findings in low-income economy countries (19). Countries typically add new payment methods to existing ones (usually P4P for different providers), replace FFS with capitation in PHC, or mix these two methods. This shift from FFS to capitation aims to contain costs, as FFS can lead to cost increases and service oversupply, jeopardizing the financial stability of purchasers (16). Capitation has been identified as a preferred approach for PHC financing in LMICs due to its potential to align incentives with population health goals (60). For instance, Thailand’s capitation-based system has helped expand comprehensive PHC coverage at the district level (60, 61). Capitation is known to promote efficiency (62), reduce costs (63, 64), generate attractive provider revenue (65), promote compliance with guidelines and policies (66), and improve provider performance and patient education (67). However, it can also affect care quality and quantity (65), may discourage providers from serving high-risk patients (68), and affect patient-provider relationships (68). Many countries have adopted mixed payment systems that combine FFS and capitation (34, 36–43, 49, 50), a strategy supported by high-income country literature in PHC (16). Mixed payment models can offset the disadvantages of pure payment methods and make them attractive options for policymakers (16, 69–73).

The factors influencing payment reforms in the surveyed countries are broadly consistent with those in the international literature (18, 19). Contextual factors such as political will and regulatory frameworks play crucial roles in most reforms, particularly when they coincide with other health policies and political priorities (19). We found that the success of a reform largely depended on its clarity and transparency in content elements such as performance indicators, payment rates and quality criteria as well as its potential to generate positive perceptions and interests among key stakeholders, particularly providers. Consistent with previous studies, deficiencies in these elements can lead to several problems, such as tensions between providers and reformers, which can stagnate reform efforts (19, 74). The reform process is often hindered by chronic underfunding, largely driven by donor influence in low-resource settings and further exacerbated by high fragmentation in financing and health service delivery systems, as consistently observed by previous researchers (19, 74). Conversely, piloting reforms is often considered a key process facilitator, helping countries identify implementation challenges and inform redesign before nationwide rollout. However, the selection of pilot sites must also consider political and contextual factors. In Ghana, for example, choosing a region associated with opposition political interests led to suspicion and resistance, illustrating how local dynamics can undermine the credibility of reform piloting (32, 58). Finally, we emphasize the importance of stakeholder engagement for successful reform implementation, but ensuring that stakeholders have sufficient capacity to carry out the assigned tasks is crucial. Many payment reforms were hindered by the lack of sufficient capacity of stakeholders (e.g., financial capacity, human resources, technical skills, and tools such as IT). An interesting approach is the technique observed in Ghana (32) of mapping the capacities of stakeholders and forming groups for those who are unable to implement reforms independently. Further research is needed to evaluate this practice and shed light on potential challenges and strengths or feasibility in other, particularly resource-limited, countries.

This study highlights important research gaps. In African Commonwealth countries, current evidence on provider payment reforms for strategic health purchasing is limited—not only because such reforms have been implemented in relatively few countries, but also because, where they do exist, the available evidence often focuses on specific types of reforms and/or presents evaluations from a single perspective. Our results help define indications for future studies. First, PBF is the most commonly implemented reform (studies were identified in six of the eight countries for which evidence was found). Previous studies have also shown that such outcome-based payment models have gained traction, but their scope is narrow and they focus on specific diseases or conditions (18, 75, 76). In particular, studies have reported mixed results concerning the long-term viability of P4P in similar settings (77, 78), and a review of PBF in LMICs concluded that no definitive conclusions could be drawn regarding the likely impact of PBF (79). Therefore, the effectiveness of these methods in the studied settings remains to be investigated. Second, it is worth noting that the majority of studies examining the impacts of capitation reforms focus on experiences in Ghana. While these insights are undoubtedly valuable, they may not provide a comprehensive understanding of how such payment reforms play out in other countries on the continent. Third, many of the reforms included are broad and target multiple providers at the same time. This approach often lacks the nuance needed to determine which methods are most effective for certain types of providers. Although some countries combine methods that can sometimes mitigate the unintended consequences of individual payment methods, previous research has suggested that certain methods may be more suitable for particular types of providers while proving ineffective for others (80). Examples include capitation for PHC plus FFS for priority interventions, FFS with P4P for episodic care, and DRGs with global budget (70). This practice of which methods should be blended for specific types of providers particularly needs to be investigated in the studied countries. Additionally, it is crucial to acknowledge the absence of experiences regarding other value-based payment methods, such as bundled payment methods, within the study settings. Bundled payments are crucial for effective care continuity, especially for chronic conditions (70). Future research should examine the potential of such payment initiatives in African settings and assess their feasibility. Additionally, the provider payment method defines the mechanism used to transfer funds from purchasers to providers (20) and is just one of five interrelated elements of the strategic purchasing framework (4, 5). Future studies could be aimed at a more comprehensive reform evaluation, e.g., from a multistakeholder perspective and/or in interconnection/relation with other elements of strategic purchasing. Finally, the issue of factors influencing payment reform success can be investigated via targeted original research, with a focus on developing policy recommendations for best practices to overcome specific barriers.

This review has several strengths. It is the first study to systematically synthesize provider payment reforms in African Commonwealth countries using a structured health policy framework. The search strategy included both peer-reviewed and gray literature. However, certain limitations should be noted. The review included only English-language literature. Although this is justified given that English is the common official language in Commonwealth countries, relevant local-language sources may have been excluded. Additionally, the study relied on publicly available published evidence, which may omit unpublished reform documentation or evaluations. Finally, in line with the scoping review methodology (22), we did not assess the quality of the included studies.

## 5 Conclusion

This study highlights a major research gap in healthcare provider payment reforms in African Commonwealth countries. The evidence shows a trend toward supplementing traditional methods with new ones, such as P4P, replacing FFS with capitation, or mixed models. Unlike high-income countries, which prioritize bundled payments for chronic diseases, African countries’ reforms often focus on specific diseases such as HIV or maternal health. Success factors in Africa are similar to those in high-income countries, but unique challenges include fragmented funding and heavy reliance on donors.

## Data Availability

The original contributions presented in the study are included in the article/Supplementary material, further inquiries can be directed to the corresponding author.
