# Supplementary material for: Health care provider payment reforms in African states of the Commonwealth—a scoping review
Source: Front Public Health. 2025 Jun 18;13:1446497. doi: 10.3389/fpubh.2025.1446497 (PMC12213801; doi:10.3389/fpubh.2025.1446497)
Supplement: Supplementary file 1 [file Data_Sheet_1.PDF]

Supplementary Material

Health care provider payment reforms in African states of the Commonwealth - a scoping review

Costase Ndayishimiye[1,2]*, Christoph Sowada[2], Katarzyna Dubas-Jakóbczyk[2]

*** Correspondance: Costase Ndayishimiye**: costase.ndayishimiye@doctoral.uj.edu.pl

Supplementary Tables:

Table S1. Search terms for databases: PubMed, Web of Science & Scopus

Healthcare provider	"healthcare provider*" OR "care provider*" OR "health provider*" OR "health service provider*" OR "health practi*" OR "healthcare practi*" OR "medical practi*" OR "health institution*" OR "healthcare institution*" OR "health care institution*" OR caregiver*
AND	
Payment	pay* OR compensat* OR incentive* OR financ* OR reimburs* OR purchas* OR reward* OR bonus*
AND	
Country	Botswana* OR Cameroon* OR Gabon* OR Gambia* OR Ghan* OR Kenya* OR Eswatini* OR Lesotho* OR Malawi* OR Mauriti* OR Mozambiqu* OR Namibia* OR Nigeria* OR Rwand* OR Seychell* OR "Sierra Leone*" OR "South Africa*" OR Togo* OR Uganda* OR Tanzania* OR Zambia*

Table S2. Search in Scopus
Search date: **01/07/2023**

**Search**	**Query**	**Hints**
#45	((TITLE-ABS-KEY (caregiver*)) OR (TITLE-ABS-KEY ("health care institution*")) OR (TITLE-ABS-KEY ("healthcare institution*")) OR (TITLE-ABS-KEY ("health institution*")) OR (TITLE-ABS-KEY ("medical practi*")) OR (TITLE-ABS-KEY ("healthcare practi*")) OR (TITLE-ABS-KEY ("health practi*")) OR (TITLE-ABS-KEY ("health service provider*")) OR (TITLE-ABS-KEY ("health provider*")) OR (TITLE-ABS-KEY ("care provider*"))) AND ((TITLE-ABS-KEY (pay*)) OR (TITLE-ABS-KEY (compensat*)) OR (TITLE-ABS-KEY (incentive*)) OR (TITLE-ABS-KEY (financ*)) OR (TITLE-ABS-KEY (reimburs*)) OR (TITLE-ABS-KEY (purchas*)) OR (TITLE-ABS-KEY (reward*)) OR (TITLE-ABS-KEY (bonus*))) AND ((TITLE-ABS-KEY (botswana*)) OR (TITLE-ABS-KEY (cameroon*)) OR (TITLE-ABS-KEY (gabon*)) OR (TITLE-ABS-	1,126

	KEY (gambia*)) OR (TITLE-ABS-KEY (ghan*)) OR (TITLE-ABS-KEY (kenya*)) OR (TITLE-ABS-KEY (eswatini*)) OR (TITLE-ABS-KEY (lesotho*)) OR (TITLE-ABS-KEY (malawi*)) OR (TITLE-ABS-KEY (mauriti*)) OR (TITLE-ABS-KEY (mozambiqu*)) OR (TITLE-ABS-KEY (namibia*)) OR (TITLE-ABS-KEY (nigeria*)) OR (TITLE-ABS-KEY (rwand*)) OR (TITLE-ABS-KEY (seychell*)) OR (TITLE-ABS-KEY ("sierra leone*")) OR (TITLE-ABS-KEY ("south africa*")) OR (TITLE-ABS-KEY (togo*)) OR (TITLE-ABS-KEY (uganda*)) OR (TITLE-ABS-KEY (tanzania*)) OR (TITLE-ABS-KEY (zambia*))) AND PUBYEAR > 2009 AND PUBYEAR < 2024 AND (LIMIT-TO (DOCTYPE , "ar") OR LIMIT-TO (DOCTYPE , "re") OR LIMIT-TO (DOCTYPE , "ch") OR LIMIT-TO (DOCTYPE , "bk")) AND (LIMIT-TO (LANGUAGE , "english"))	
#44	((TITLE-ABS-KEY (caregiver*)) OR (TITLE-ABS-KEY ("health care institution*")) OR (TITLE-ABS-KEY ("healthcare institution*")) OR (TITLE-ABS-KEY ("health institution*")) OR (TITLE-ABS-KEY ("medical practi*")) OR (TITLE-ABS-KEY ("healthcare practi*")) OR (TITLE-ABS-KEY ("health practi*")) OR (TITLE-ABS-KEY ("health service provider*")) OR (TITLE-ABS-KEY ("health provider*")) OR (TITLE-ABS-KEY ("care provider*"))) AND ((TITLE-ABS-KEY (pay*)) OR (TITLE-ABS-KEY (compensat*)) OR (TITLE-ABS-KEY (incentive*)) OR (TITLE-ABS-KEY (financ*)) OR (TITLE-ABS-KEY (reimburs*)) OR (TITLE-ABS-KEY (purchas*)) OR (TITLE-ABS-KEY (reward*)) OR (TITLE-ABS-KEY (bonus*))) AND ((TITLE-ABS-KEY (botswana*)) OR (TITLE-ABS-KEY (cameroon*)) OR (TITLE-ABS-KEY (gabon*)) OR (TITLE-ABS-KEY (gambia*)) OR (TITLE-ABS-KEY (ghan*)) OR (TITLE-ABS-KEY (kenya*)) OR (TITLE-ABS-KEY (eswatini*)) OR (TITLE-ABS-KEY (lesotho*)) OR (TITLE-ABS-KEY (malawi*)) OR (TITLE-ABS-KEY (mauriti*)) OR (TITLE-ABS-KEY (mozambiqu*)) OR (TITLE-ABS-KEY (namibia*)) OR (TITLE-ABS-KEY (nigeria*)) OR (TITLE-ABS-KEY (rwand*)) OR (TITLE-ABS-KEY (seychell*)) OR (TITLE-ABS-KEY ("sierra leone*")) OR (TITLE-ABS-KEY ("south africa*")) OR (TITLE-ABS-KEY (togo*)) OR (TITLE-ABS-KEY (uganda*)) OR (TITLE-ABS-KEY (tanzania*)) OR (TITLE-ABS-KEY (zambia*)))	1,510
#43	(TITLE-ABS-KEY (botswana*)) OR (TITLE-ABS-KEY (cameroon*)) OR (TITLE-ABS-KEY (gabon*)) OR (TITLE-ABS-KEY (gambia*)) OR (TITLE-ABS-KEY (ghan*)) OR (TITLE-ABS-KEY (kenya*)) OR (TITLE-ABS-KEY (eswatini*)) OR (TITLE-ABS-KEY (lesotho*)) OR (TITLE-ABS-KEY (malawi*)) OR (TITLE-ABS-KEY (mauriti*)) OR (TITLE-ABS-KEY (mozambiqu*)) OR (TITLE-ABS-KEY (namibia*)) OR (TITLE-ABS-KEY (nigeria*)) OR (TITLE-ABS-KEY (rwand*)) OR (TITLE-ABS-KEY (seychell*)) OR (TITLE-ABS-KEY ("sierra leone*")) OR (TITLE-ABS-KEY ("south africa*")) OR (TITLE-ABS-KEY (togo*)) OR (TITLE-ABS-KEY (uganda*)) OR (TITLE-ABS-KEY (tanzania*)) OR (TITLE-ABS-KEY (zambia*))	627,729
#42	TITLE-ABS-KEY (zambia*)	16,680
#41	TITLE-ABS-KEY (tanzania*)	41,902
#40	TITLE-ABS-KEY (uganda*)	39,305
#39	TITLE-ABS-KEY (togo*)	4,899

#38	TITLE-ABS-KEY ("south africa*")	219,969
#37	TITLE-ABS-KEY ("sierra leone*")	7,887
#36	TITLE-ABS-KEY (seychell*)	3,233
#35	TITLE-ABS-KEY (rwand*)	12,516
#34	TITLE-ABS-KEY (nigeria*)	122,564
#33	TITLE-ABS-KEY (namibia*)	12,353
#32	TITLE-ABS-KEY (mozambiqu*)	14,557
#31	TITLE-ABS-KEY (mauriti*)	9,685
#30	TITLE-ABS-KEY (malawi*)	18,126
#29	TITLE-ABS-KEY (lesotho*)	3,735
#28	TITLE-ABS-KEY (eswatini*)	675
#27	TITLE-ABS-KEY (kenya*)	62,653
#26	TITLE-ABS-KEY (ghan*)	47,008
#25	TITLE-ABS-KEY (gambia*)	13,101
#24	TITLE-ABS-KEY (gabon*)	6,219
#23	TITLE-ABS-KEY (cameroon*)	22,418
#22	TITLE-ABS-KEY (botswana*)	11,339
#21	(TITLE-ABS-KEY (pay*)) OR (TITLE-ABS-KEY (compensat*)) OR (TITLE-ABS-KEY (incentive*)) OR (TITLE-ABS-KEY (financ*)) OR (TITLE-ABS-KEY (reimburs*)) OR (TITLE-ABS-KEY (purchas*)) OR (TITLE-ABS-KEY (reward*)) OR (TITLE-ABS-KEY (bonus*))	2,535,112
#20	TITLE-ABS-KEY (bonus*)	10,441
#19	TITLE-ABS-KEY (reward*)	168,224
#18	TITLE-ABS-KEY (purchas*)	207,883
#17	TITLE-ABS-KEY (reimburs*)	78,817
#16	TITLE-ABS-KEY (financ*)	895,829
#15	TITLE-ABS-KEY (incentive*)	184,348
#14	TITLE-ABS-KEY (compensat*)	702,771
#13	TITLE-ABS-KEY (pay*)	531,025
#12	(TITLE-ABS-KEY (caregiver*)) OR (TITLE-ABS-KEY ("health care institution*")) OR (TITLE-ABS-KEY ("healthcare institution*")) OR (TITLE-ABS-KEY ("health institution*")) OR (TITLE-ABS-KEY ("medical practi*")) OR (TITLE-ABS-KEY ("healthcare practi*")) OR (TITLE-ABS-KEY ("health practi*")) OR (TITLE-ABS-KEY ("health service provider*")) OR (TITLE-ABS-KEY ("health provider*")) OR (TITLE-ABS-KEY ("care provider*"))	446,077
#11	TITLE-ABS-KEY (caregiver*)	155,338
#10	("health care institution*")	4,830
#9	TITLE-ABS-KEY ("healthcare institution*")	4,853
#8	TITLE-ABS-KEY ("health institution*")	7,613
#7	TITLE-ABS-KEY ("MEDICAL PRACTI*")	129,695
#6	TITLE-ABS-KEY ("HEALTHCARE PRACTI*")	6,538
#5	TITLE-ABS-KEY ("HEALTH PRACTI*")	57,369
#4	TITLE-ABS-KEY ("HEALTH SERVICE PROVIDER*")	2,511
#3	TITLE-ABS-KEY ("HEALTH PROVIDER*")	11,237
#2	TITLE-ABS-KEY ("CARE PROVIDER*")	83,732
#1	TITLE-ABS-KEY ("healthcare provider*")	41,295

Table S3. Search in PubMed
Search date: **28/06/2023**

Search	Query	Hints
#45	(("healthcare provider*"[Title/Abstract] OR "care provider*"[Title/Abstract] OR "health provider*"[Title/Abstract] OR "health service provider*"[Title/Abstract] OR "health practic*"[Title/Abstract] OR "healthcare practic*"[Title/Abstract] OR "medical practi*"[Title/Abstract] OR "health institution*"[Title/Abstract] OR "healthcare institution*"[Title/Abstract] OR "health care institution*"[Title/Abstract] OR "caregiver*"[Title/Abstract]) AND ("pay"[Title/Abstract] OR "compensat*"[Title/Abstract] OR "incentive*"[Title/Abstract] OR "financ*"[Title/Abstract] OR "reimburs*"[Title/Abstract] OR "purchas*"[Title/Abstract] OR "reward*"[Title/Abstract] OR "bonus*"[Title/Abstract]) AND ("botswana*"[Title/Abstract] OR "cameroon*"[Title/Abstract] OR "gabon*"[Title/Abstract] OR "gambia*"[Title/Abstract] OR "ghan*"[Title/Abstract] OR "kenya*"[Title/Abstract] OR "eswatini*"[Title/Abstract] OR "lesotho*"[Title/Abstract] OR "malawi*"[Title/Abstract] OR "mauriti*"[Title/Abstract] OR "mozambiqu*"[Title/Abstract] OR "namibia*"[Title/Abstract] OR "nigeria*"[Title/Abstract] OR "rwand*"[Title/Abstract] OR "seychell*"[Title/Abstract] OR "sierra leone*"[Title/Abstract] OR "south africa*"[Title/Abstract] OR "togo*"[Title/Abstract] OR "uganda*"[Title/Abstract] OR "tanzania*"[Title/Abstract] OR "zambia*"[Title/Abstract])) AND ((english[Filter]) AND (2010:2023[pdat]))	841
#44	("healthcare provider*"[Title/Abstract] OR "care provider*"[Title/Abstract] OR "health provider*"[Title/Abstract] OR "health service provider*"[Title/Abstract] OR "health practic*"[Title/Abstract] OR "healthcare practic*"[Title/Abstract] OR "medical practi*"[Title/Abstract] OR "health institution*"[Title/Abstract] OR "healthcare institution*"[Title/Abstract] OR "health care institution*"[Title/Abstract] OR "caregiver*"[Title/Abstract]) AND ("pay"[Title/Abstract] OR "compensat*"[Title/Abstract] OR "incentive*"[Title/Abstract] OR "financ*"[Title/Abstract] OR "reimburs*"[Title/Abstract] OR "purchas*"[Title/Abstract] OR "reward*"[Title/Abstract] OR "bonus*"[Title/Abstract]) AND ("botswana*"[Title/Abstract] OR "cameroon*"[Title/Abstract] OR "gabon*"[Title/Abstract] OR "gambia*"[Title/Abstract] OR "ghan*"[Title/Abstract] OR "kenya*"[Title/Abstract] OR "eswatini*"[Title/Abstract] OR "lesotho*"[Title/Abstract] OR "malawi*"[Title/Abstract] OR "mauriti*"[Title/Abstract] OR "mozambiqu*"[Title/Abstract] OR "namibia*"[Title/Abstract] OR "nigeria*"[Title/Abstract] OR "rwand*"[Title/Abstract] OR "seychell*"[Title/Abstract] OR "sierra leone*"[Title/Abstract] OR "south africa*"[Title/Abstract] OR "togo*"[Title/Abstract] OR "uganda*"[Title/Abstract] OR "tanzania*"[Title/Abstract] OR "zambia*"[Title/Abstract])	969
#43	"botswana*"[Title/Abstract] OR "cameroon*"[Title/Abstract] OR "gabon*"[Title/Abstract] OR "gambia*"[Title/Abstract] OR "ghan*"[Title/Abstract] OR "kenya*"[Title/Abstract] OR "eswatini*"[Title/Abstract] OR "lesotho*"[Title/Abstract] OR "malawi*"[Title/Abstract] OR "mauriti*"[Title/Abstract] OR "mozambiqu*"[Title/Abstract] OR "namibia*"[Title/Abstract] OR "nigeria*"[Title/Abstract] OR "rwand*"[Title/Abstract] OR "seychell*"[Title/Abstract] OR "sierra	215,457

	leone*"[Title/Abstract] OR "south africa*"[Title/Abstract] OR "togo*"[Title/Abstract] OR "uganda*"[Title/Abstract] OR "tanzania*"[Title/Abstract] OR "zambia*"[Title/Abstract]	
#42	"zambia*"[Title/Abstract]	7,286
#41	"tanzania*"[Title/Abstract]	17,208
#40	"uganda*"[Title/Abstract]	20,320
#39	"togo*"[Title/Abstract]	2,071
#38	"south africa*"[Title/Abstract]	54,868
#37	"sierra leone*"[Title/Abstract]	2,904
#36	"seychell*"[Title/Abstract]	960
#35	"rwand*"[Title/Abstract]	4,354
#34	"nigeria*"[Title/Abstract]	42,863
#33	"namibia*"[Title/Abstract]	2,177
#32	"mozambiqu*"[Title/Abstract]	4,581
#31	"mauriti*"[Title/Abstract]	2,174
#30	"malawi*"[Title/Abstract]	9,463
#29	"lesotho*"[Title/Abstract]	939
#28	"eswatini*"[Title/Abstract]	333
#27	"kenya*"[Title/Abstract]	25,182
#26	"ghan*"[Title/Abstract]	16,287
#25	"gambia*"[Title/Abstract]	9,065
#24	"gabon*"[Title/Abstract]	2,483
#23	"cameroon*"[Title/Abstract]	9,194
#22	"botswana*"[Title/Abstract]	3,035
#21	"pay"[Title/Abstract] OR "compensat*"[Title/Abstract] OR "incentive*"[Title/Abstract] OR "financ*"[Title/Abstract] OR "reimburs*"[Title/Abstract] OR "purchas*"[Title/Abstract] OR "reward*"[Title/Abstract] OR "bonus*"[Title/Abstract]	514,994
#20	"bonus*"[Title/Abstract]	1,903
#19	"reward*"[Title/Abstract]	66,519
#18	"purchas*"[Title/Abstract]	38,494
#17	"reimburs*"[Title/Abstract]	32,768
#16	"financ*"[Title/Abstract]	142,544
#15	"incentive*"[Title/Abstract]	36,570
#14	"compensat*"[Title/Abstract]	184,472
#13	"pay*"[Title/Abstract]	51,075
#12	"healthcare provider*"[Title/Abstract] OR "care provider*"[Title/Abstract] OR "health provider*"[Title/Abstract] OR "health service provider*"[Title/Abstract] OR "health practic*"[Title/Abstract] OR "healthcare practic*"[Title/Abstract] OR "medical practi*"[Title/Abstract] OR "health institution*"[Title/Abstract] OR "healthcare institution*"[Title/Abstract] OR "health care institution*"[Title/Abstract] OR "caregiver*"[Title/Abstract]	247,905
#11	"caregiver*"[Title/Abstract]	91,489
#10	"health care institution*"[Title/Abstract]	3,286
#9	"healthcare institution*"[Title/Abstract]	3,066
#8	"health institution*"[Title/Abstract]	4,897
#7	"medical practi*"[Title/Abstract]	31,004
#6	"healthcare practi*"[Title/Abstract]	1,830

#5	"health practi*"[Title/Abstract]	8,515
#4	"health service provider*"[Title/Abstract]	1,549
#3	"health provider*"[Title/Abstract]	9,055
#2	"care provider*"[Title/Abstract]	69,991
#1	"healthcare provider*"[Title/Abstract]	34,676

Table S4. Search in Web of Science
Search date: **29/06/2023**

Search	Query	Hints
#45	#12 AND #21 AND #43 and 2023 or 2022 or 2021 or 2020 or 2019 or 2018 or 2017 or 2016 or 2015 or 2014 or 2013 or 2012 or 2011 or 2010 (Publication Years) and Meeting Abstract or Correction or Editorial Material or Proceeding Paper (Exclude – Document Types) and English (Languages)	960
#44	#12 AND #21 AND #43	1,086
#43	#22 OR #23 OR #24 OR #25 OR #26 OR #27 OR #28 OR #29 OR #30 OR #31 OR #32 OR #33 OR #34 OR #35 OR #36 OR #37 OR #38 OR #39 OR #40 OR #41 OR #42	486,383
#42	(TI=(Zambia*)) OR AB=(Zambia*)	13,714
#41	(TI=(Tanzania*)) OR AB=(Tanzania*)	33,265
#40	(TI=(Uganda*)) OR AB=(Uganda*)	32,390
#39	(TI=(Togo*)) OR AB=(Togo*)	3,580
#38	(TI=(“South Africa*”)) OR AB=(“South Africa*”)	165,024
#37	(TI=(“Sierra Leone*”)) OR AB=(“Sierra Leone*”)	6,183
#36	(TI=(Seychell*)) OR AB=(Seychell*)	2,622
#35	(TI=(Rwand*)) OR AB=(Rwand*)	9,819
#34	(TI=(Nigeria*)) OR AB=(Nigeria*)	82,167
#33	(TI=(Namibia*)) OR AB=(Namibia*)	9,756
#32	(TI=(Mozambiqu*)) OR AB=(Mozambiqu*)	11,699
#31	TI=(Mauriti*)) OR AB=(Mauriti*)	6,795
#30	(TI=(Malawi*)) OR AB=(Malawi*)	15,587
#29	(TI=(Lesotho*)) OR AB=(Lesotho*)	2,711
#28	(TI=(Eswatini*)) OR AB=(Eswatini*)	540
#27	(TI=(Kenya*)) OR AB=(Kenya*)	49,966
#26	(TI=(Ghan*)) OR AB=(Ghan*)	38,007
#25	(TI=(Gambia*)) OR AB=(Gambia*)	10,482
#24	(TI=(Gabon*)) OR AB=(Gabon*)	4,524
#23	(TI=(Cameroon*)) OR AB=(Cameroon*)	18,101
#22	(TI=(Botswana*)) OR AB=(Botswana*)	8,485
#21	#13 OR #14 OR #15 OR #16 OR #17 OR #18 OR #19 OR #20	1,638,766
#20	(TI=(bonus*)) OR AB=(bonus*)	7,006
#19	(TI=(reward*)) OR AB=(reward*)	120,582
#18	(TI=(purchas*)) OR AB=(purchas*)	125,304
#17	(TI=(reimburs*)) OR AB=(reimburs*)	28,692
#16	(TI=(financ*)) OR AB=(financ*)	532,036
#15	(TI=(incentive*)) OR AB=(incentive*)	130,924
#14	(TI=(compensat*)) OR AB=(compensat*)	475,434

#13	(TI=(pay*)) OR AB=(pay*)	356,588
#12	#11 OR #10 OR #9 OR #8 OR #7 OR #6 OR #5 OR #4 OR #3 OR #2 OR #1	260,268
#11	(TI=(caregiver*)) OR AB=(caregiver*)	104,584
#10	(TI=("health care institution*")) OR AB=("health care institution*")	2,824
#9	(TI=("healthcare institution*")) OR AB=("healthcare institution*")	3,345
#8	(TI=("health institution*")) OR AB=("health institution*")	4,949
#7	(TI=("medical practi*")) OR AB=("medical practi*")	28,628
#6	(TI=("healthcare practi*")) OR AB=("healthcare practi*")	4,726
#5	(TI=("health practi*")) OR AB=("health practi*")	15,661
#4	(TI=("health service provider*")) OR AB=("health service provider*")	1,767
#3	(TI=("health provider*")) OR AB=("health provider*")	8,888
#2	(TI=("care provider*")) OR AB=("care provider*")	62,863
#1	(TI=("healthcare provider*")) OR AB=("healthcare provider*")	33,568

Table S5. Google Engine Search
Date of search: 01/07/2023

i) Procedure:
Similar to scientific databases, we searched Google Engine using key terms. However, Google Engine limits searches to 32 words. We used the most appropriate keywords and combined some of them. In addition, Google Engine does not index the publications by abstract. Therefore, the command was adapted to search for the exact terms in the "title" or in the "text". The corresponding commands was also applied: |= OR, &= AND. Since Google Engine can index files in different formats, we also added a command to limit the file types to those that are common in scientific formats and can be imported into our citation manager (Mendeley). The file types were limited to (.pdf) and TeX/LaTeX (.tex).
The final search query was as follows:
intitle | intext:"provider payment" | "strategic purchasing" and Botswana | Cameroon | Gabon | Gambia | Ghana | Kenya | Eswatini | Lesotho | Malawi | Mauritius | Mozambique | Namibia | Nigeria | Rwanda | Seychelles | "Sierra Leone" | "South Africa" | Togo | Uganda | Tanzania | Zambia filetype:pdf

ii) Search results:
We allowed Google Engine to omit entries that were very similar to the results already displayed. As a result, the most relevant results were displayed: 103 publications on 11 pages. They were all taken into account.

Table S6. List of hand-searched organizations, their websites and search dates.

Organization name	Website/Link	Search date
Strategic Purchasing Africa Resource Centre (SPARC)	https://sparc.africa/	07/2023
WHO via WHO African Region	https://www.afro.who.int/	07/2023
Responsive and Resilient Health Systems (RESYST)	https://resyst.lshtm.ac.uk/resources	07/2023
Health Finance and Governance (HFG) country publications	https://www.hfgproject.org/hfg-country-final-reports/	07/2023
World Bank	https://www.worldbank.org/en/topic/health	07/2023

Table S7. List of inclusion and exclusion criteria for studies.

**Inclusion**	**Exclusion**
-Studies focused on healthcare provider payment and strategic purchasing.<br>- Peer-reviewed empirical studies, policy-briefs, theoretical papers, technical reports, books/chapters, theses.<br>-Published between 2013 and 2023<br>-Full text in English.<br>-African Commonwealth countries only	-Publications that do not focus on health care providers (e.g., social insurance schemes, community financing, health cards or voucher systems, cost recovery, subsidized payment schemes)<br>-Wrong publication types (conference abstract, commentary, letters to the editor, erratum)<br>- Full text is in other languages.<br>- Other countries (non-Commonwealth countries within or outside Africa, Commonwealth countries outside Africa)
